# A comparison of performance of Deca Iron and Triple Deca Iron ultra-triathletes

**DOI:** 10.1186/2193-1801-3-461

**Published:** 2014-08-24

**Authors:** Beat Knechtle, Thomas Rosemann, Romuald Lepers, Christoph Alexander Rüst

**Affiliations:** Institute of Primary Care, University of Zurich, Zurich, Switzerland; Gesundheitszentrum St. Gallen, Vadianstrasse 26, 9001 St. Gallen, Switzerland; INSERM U1093, Faculty of Sport Sciences, University of Burgundy, Dijon, France

**Keywords:** Triathlon, Swimming, Cycling, Running, Ultra-endurance

## Abstract

This study intended to compare the performance of ultra-triathletes competing in a Deca Iron ultra-triathlon (*i.e.* 10 times 3.8 km swimming, 180 km cycling, and 42.2 km running) with the performance of athletes competing in a Triple Deca Iron ultra-triathlon (*i.e.* 30 times 3.8 km swimming, 180 km cycling, and 42.2 km running). Split and overall race times of six male finishers in a Deca Iron ultra-triathlon and eight male finishers in a Triple Deca Iron ultra-triathlon were analysed using multiple *t*-tests, linear and non-linear regression analyses, and analysis of variance. Among the 19 starters (*i.e.* 17 men and two women) in the Deca Iron ultra-triathlon, six men (*i.e.* 35.3% of all starters) finished the race. The mean swimming, cycling, running and overall race times of the six finishers across the ten days were 1:19 ± 0:09 h:min, 6:36 ± 0:19 h:min, 6:03 ± 0:47 h:min and 14:44 ± 1:17 h:min, respectively. The times of the split disciplines and overall race time increased linearly across the ten days. Total transition times did not change significantly across the days and were equals to 48 ± 8 min. Among the 22 starters (*i.e.* 20 men and two women) in the Triple Deca Iron ultra-triathlon, eight men (*i.e.* 36.4% of all starters) finished. The mean swimming, cycling, running and overall race times of the eight finishers across the 30 days were 1:11 ± 0:07 h:min, 6:19 ± 0:32 h:min, 5:34 ± 1:15 h:min and 13:44 ± 1:50 h:min, respectively. Split and overall race times showed no change across the 30 days. Total transition times showed no change across the days and were equal to 41 ± 11 min. To summarize, the daily performance decreased across the ten days for the Deca Iron ultra-triathletes (*i.e.* positive pacing) while it remained unchanged across the 30 days for the Triple Deca Iron ultra-triathletes (*i.e.* even pacing).

## Background

Ultra-endurance performance is defined as any endurance performance of six hours or longer in duration (Zaryski and Smith 2005). An Ironman triathlon covering 3.8 km swimming, 180 km cycling and 42.2 km running with the fastest winner times of ~8 hrs has to be considered as an ultra-endurance performance (Lepers 2008). Apart from the classical Ironman triathlon held as a single stage race, also longer ultra-triathlons such as multi-stage races with the completion of consecutive Ironman triathlons held for several days are known (Herbst et al. 2011; Knechtle et al. 2011a).

In a Deca Iron ultra-triathlon - where each day an Ironman triathlon has to be finished for ten consecutive days - performance decreased with increasing duration of the race (Herbst et al. 2011; Knechtle et al. 2012a) where the fastest Ironman was achieved on the first day (Herbst et al. 2011; Knechtle et al. 2008a) and the slowest on the last day (Herbst et al. 2011). A multi-stage ultra-triathlon such as a Deca Iron ultra-triathlon is a highly selective race and less than 50% of the starters are able to finish (Herbst et al. 2011; Knechtle et al. 2012a). The most important predictor variables for a successful finish in a Deca Iron ultra-triathlon were extensive previous experience since the number of finished Triple Iron ultra-triathlons and the personal best time in a Triple Iron ultra-triathlon were related to overall race time (Herbst et al. 2011).

Since the first edition of a Deca Iron ultra-triathlon in 2006 (Knechtle et al. 2008a), several races of this kind have been held mainly in Monterrey, Mexico (Herbst et al. 2011; Knechtle et al. 2012a). However, in autumn 2013, ultra-endurance triathletes in Lonato des Garda, Italy, intended to go for new limits by organizing a Triple Deca Iron ultra-triathlon, where the athletes had to finish each day an Ironman triathlon for 30 consecutive days. In the same race, a second group of athletes competed in a separate Deca Iron ultra-triathlon to finish ten Ironman triathlons within ten consecutive days.

Little is known about the pacing strategy in ultra-endurance performance (Abbiss and Laursen 2008). Actual evidence suggests that during endurance and ultra-endurance performance well-trained athletes tend to adopt a positive pacing strategy, whereby after peak speed is reached, the athlete progressively slows (Abbiss and Laursen 2008). The underlying mechanisms influencing the regulation of pace during exercise are currently unclear (Abbiss and Laursen 2008). It has been suggested, however, that self-selected exercise intensity is regulated within the brain based on a complex algorithm involving peripheral sensory feedback and the anticipated workload remaining (St Clair Gibson et al. 2006).

To date, to the best of our knowledge, the Triple Deca Iron ultra-triathlon represents the longest event ever finished by triathletes combining swimming, cycling and running. However, in summer 2013, an athlete completed in Laval, Québec, Canada, for the first time in history in a self-paced race the total distance of 33 Ironman triathlons within 33 consecutive days (Knechtle et al. 2014). The athlete finished the total distance of 7,458 km (*i.e.* 125 km swimming, 5,940 km cycling and 1,393 km running) within 410 h and finished each Ironman triathlon in a mean time of 12:27 h:min. During the 33 days, the athlete became slower in swimming, transition time 1, and transition time 2. However, in cycling, running and overall race time, the athlete was able to maintain his performance during the 33 days (Knechtle et al. 2014). The question is now whether athletes competing in an official race covering 30 Ironman triathlons within 30 days would be able to maintain their performance during one month as this athlete showed in 33 days in his self-paced race. The aim of the present study was therefore to compare the changes in performance over days for both Triple Deca Iron ultra-triathletes and Deca Iron ultra-triathletes. Based upon previous findings in field studies for Deca Iron ultra-triathletes and in the case study with the 33 Ironman triathlons it was hypothesized that performance would decrease in Deca Iron ultra-triathletes, but not in Triple Deca Iron ultra-triathletes.

## Methods

### Ethics

This study was approved by the Institutional Review Board of St. Gallen, Switzerland, with a waiver of the requirement for informed consent given that the study involved the analysis of publicly available data.

### The races

The Deca Iron ultra-triathlon race consisted in performing one Ironman distance triathlon (*i.e.* 3.8 km swimming, 180 km cycling and 42 km running) daily for ten consecutive days whereas the Triple Deca Iron ultra-triathlon consisted in performing one Ironman distance triathlon daily for 30 consecutive days. The races were held in and around ‘Parco La Quiete’ (http://www.parcolaquiete.it) in Lonato del Garda in the North of Italy and south to Lake Garda. The Triple Deca Iron ultra-triathlon started on September 8th, 2013, and the Deca Iron ultra-triathlon started 20 days later on September 28th, 2013. Swimming was held in a non-heated 25-m out-door pool where wetsuits were allowed. Cycling was performed as a 180-km non-drafting time trial on open roads in the hilly surroundings near ‘Parco La Quiete’ on laps of 6 km. Running was held around the lake in ‘Parco La Quiete’ on flat laps of one km on grass (~50%) and stone slabs (~50%). Laps in swimming were counted manually by the staff of the race direction. Laps in cycling and running were counted electronically by using a chip system. On Day 7, cycling laps were counted manually due to problems with the electronic chip system. In the first 21 days, air temperature was at ~25–30°C and water temperature at ~25°C. In the last nine days, weather conditions changed considerably where air temperature continuously dropped to ~14°C and water temperature dropped to ~17°C by the end of the race. In the last three days of the race, rain was continuously falling.

### Data collection and data analyses

The data set for this study was obtained from the race director for electronically recorded split times. Overall race times and split times (*i.e.* 3.8 km swimming, 180 km cycling and 42 km running) of all female and male starters were analysed regarding changes over days. Each set of data was tested for normal distribution (D’Agostino and Pearson omnibus normality test) and for homogeneity of variances (Levene’s test) before statistical analyses. Since the change in sex difference in endurance is assumed to be non-linear (Reinboud 2004), we calculated both the linear and the non-linear regression models that fit the data best. We compared the best-fit non-linear models to the linear models using Akaike’s Information Criteria (AIC) as well as F-test in order to show whether the linear or the non-linear model would be the most appropriate to explain the trend of the data. Differences in absolute and relative performance between finishers in the Deca Iron ultra-triathlon and in the Triple Deca Iron ultra-triathlon were compared using multiple *t*-test analyses with individual analysis of each pair of Deca and Triple Deca Iron ultra-triathletes and with Holm-Sidak correction for multiple comparisons. Absolute and relative performance of the Deca Iron ultra-triathletes were compared with the performance of Day 1–10, Day 11–20 and Day 21–30 of the Triple Deca Iron ultra-triathletes using repeated measures one-way analysis of variance (ANOVA) with the Greenhouse-Geisser correction and Tukey’s multiple comparison test with individual variances computed for each comparison. Statistical analyses were performed using CurveExpert Professional (Version 2.0.3, Hyams D.G.) and GraphPad Prism (Version 6.01, GraphPad Software, La Jolla, CA, USA). Significance was accepted at *p* < 0.05 (two-sided for *t*-tests). Data in the text and figures are given as mean ± standard deviation (SD).

## Results

### Deca iron ultra-triathlon

**Figure 1:**
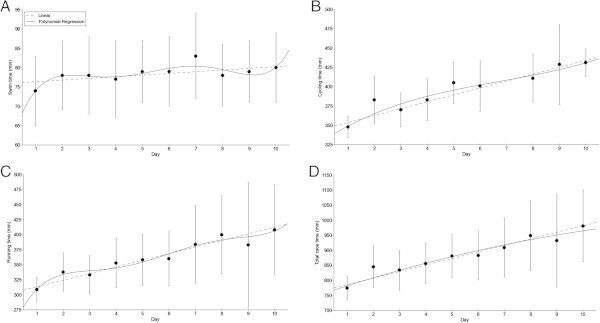
**Changes in split and overall race times during the Deca Iron ultra-triathlon. (Panel A)** swimming, **(Panel B)** cycling, **(Panel C)** running, **(Panel D)** overall race times (*n* = 6). Some cycling times on day 7 were not available. Values are means ± SD.

Among the 19 starters (*i.e.* 17 men and two women), six men (*i.e.* 35.3% of all starters) finished the race. Among the non-finishers, six triathletes stopped before the 6th day and seven triathletes stopped between the 6th and the 9th day. The mean swimming, cycling, running and overall race times of the six finishers across the ten days were 1:18 ± 0:08 h:min, 6:35 ± 0:37 h:min, 6:02 ± 1:00 h:min and 14:44 ± 1:44 h:min, respectively. All split times and overall race times increased linearly across the ten days (Figure 1). Total transition times did not change significantly across the days and were equals to 48 ± 8 min. The overall race time of the winner (*i.e.* the triathlete who had the fastest overall time after the ten days) was 129:33 h:min. His mean swimming, cycling, running and overall race times across the ten days were 1:06 ± 0:01 h:min, 6:14 ± 0:25 h:min, 5:06 ± 0:16 h:min and 12:57 ± 0:35 h:min, respectively.

### Triple Deca iron ultra-triathlon

**Figure 2:**
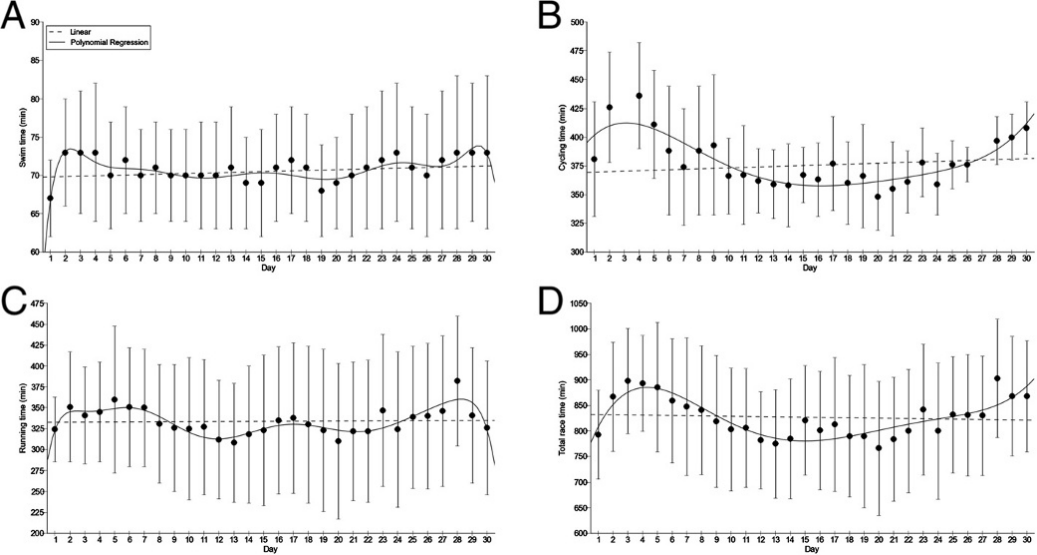
**Changes in split and overall race times during the Triple Deca Iron ultra-triathlon. (Panel A)** swimming, **(Panel B)** cycling, **(Panel C)** running, **(Panel D)** overall race times (*n* = 8). Some cycling times on day 3 and 27 were not available. Values are means ± SD.

Among the 22 starters (*i.e.* 20 men and two women), eight men (*i.e.* 36.4% of all starters) finished the 30 Ironman distance triathlon. Among the non-finishers, six triathletes finished between one and ten Ironman distances, five triathletes finished between 11 and 20 Ironman distances and three triathletes finished between 21 and 29 Ironman distances. The mean swimming, cycling, running and overall race times of the eight successful finishers across the 30 days were 1:10 ± 0:07 h:min, 6:18 ± 0:42 h:min, 5:33 ± 1:17 h:min and 13:46 ± 1:57 h:min, respectively. In contrast to the Deca Iron ultra-triathlon, the times of the different disciplines and the total time did not change across the 30 days (Figure 2). The changes were non-linearly in swimming (*i.e.* polynomial regression 10th degree), cycling (*i.e.* polynomial regression 5th degree), running (*i.e.* polynomial regression 10th degree), and overall race time (*i.e.* polynomial regression 5th degree). Total transition times did not change significantly across the days and were equal to 41 ± 11 min. The overall race time of the winner (*i.e.* the triathlete who had the fastest overall race time after the 30 events) was 356:33 h:min. His mean swimming, cycling, running and overall race times across the 30 days were 1:12 ± 0:03 h:min, 6:12 ± 0:29 h:min, 3:56 ± 0:20 h:min and 11:53 ± 0:46 h:min, respectively.

### Comparison between the 10 days in the Deca with the 3 × 10 days in the Triple Deca

**Figure 3:**
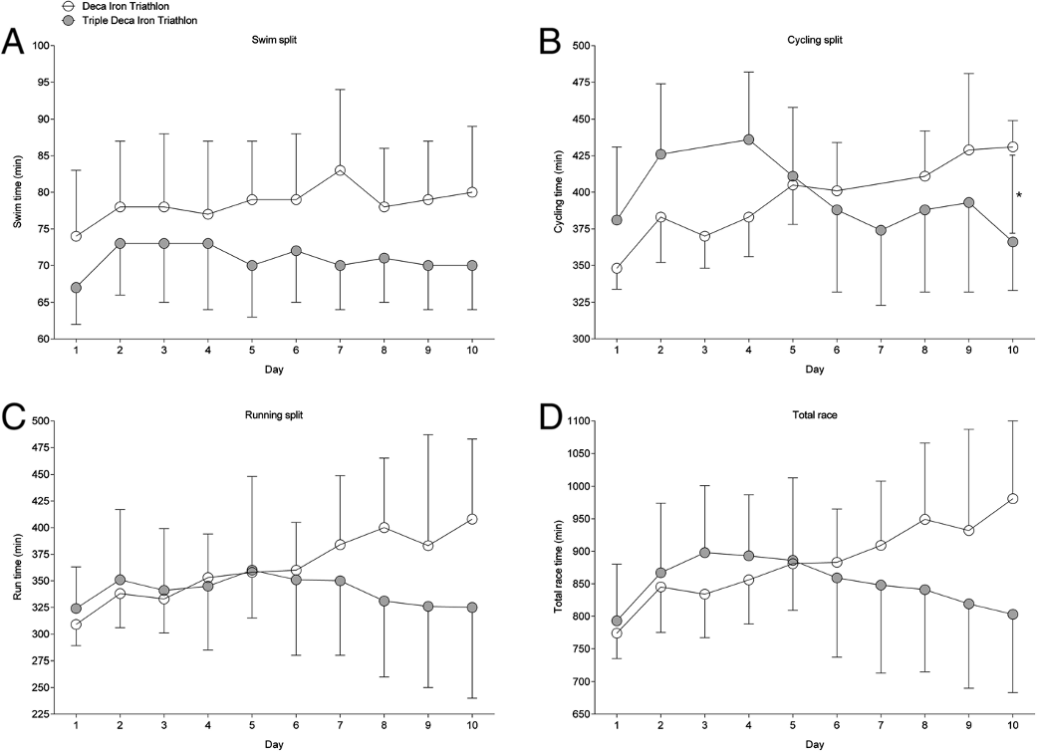
**Comparison of split and overall race times for Deca Iron ultra-triathletes (** ***i.e.*** **all ten race days) and Triple Deca Iron ultra-triathletes (** ***i.e.*** **Day 1-Day 10) expressed in absolute race times. (Panel A)** swimming, **(Panel B)** cycling, **(Panel C)** running, **(Panel D)** overall race times. Values are means ± SD.

**Figure 4:**
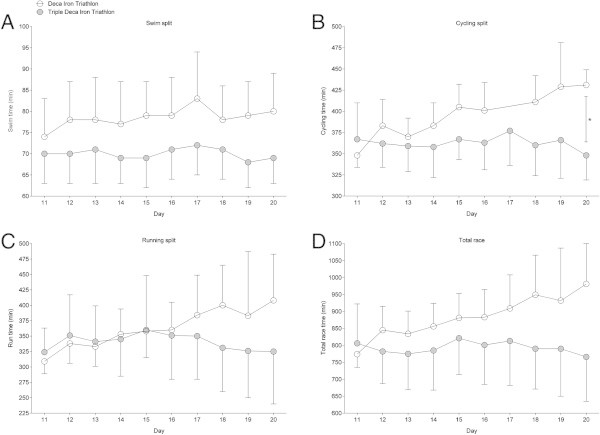
**Comparison of split and overall race times for Deca Iron ultra-triathletes (** ***i.e.*** **all ten race days) and Triple Deca Iron ultra-triathletes (** ***i.e.*** **Day 11-Day 20) expressed in absolute race times. (Panel A)** swimming, **(Panel B)** cycling, **(Panel C)** running, **(Panel D)** overall race times. Values are means ± SD.

**Figure 5:**
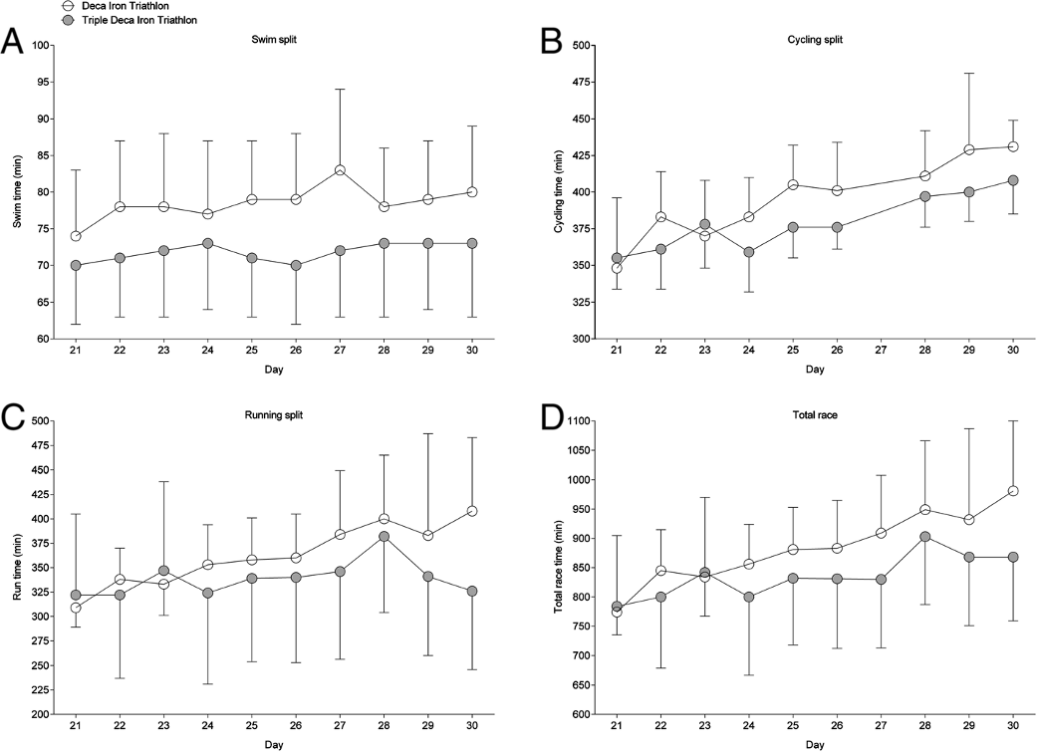
**Comparison of split and overall race times for Deca Iron ultra-triathletes (** ***i.e.*** **all ten race days) and Triple Deca Iron ultra-triathletes (** ***i.e.*** **Day 21-Day 30) expressed in absolute race times. (Panel A)** swimming, **(Panel B)** cycling, **(Panel C)** running, **(Panel D)** overall race times. Values are means ± SD.

Figure 3 presents the comparison of swimming, cycling, running, and overall race times for the Deca Iron ultra-triathletes with the first ten days of the Triple Deca Iron ultra-triathletes expressed in absolute race times. There were no differences in swimming (Figure 3A) and running (Figure 3C), but Triple Deca Iron ultra-triathletes were faster in cycling (Figure 3B) on Day 10 than Deca Iron ultra-triathletes. Figure 4 presents the same comparisons for Day 11–20 in the Triple Deca Iron ultra-triathlon with Day 1–10 in the Deca Iron ultra-triathlon. Similarly, the Triple Deca Iron ultra-triathletes were faster in cycling (Figure 4B) on Day 10 than the Deca Iron ultra-triathletes. Figure 5 compares Day 21–30 in the Triple Deca Iron ultra-triathlon with Day 1–10 in the Deca Iron ultra-triathlon. There were no differences in the performance between the finishers.

**Figure 6:**
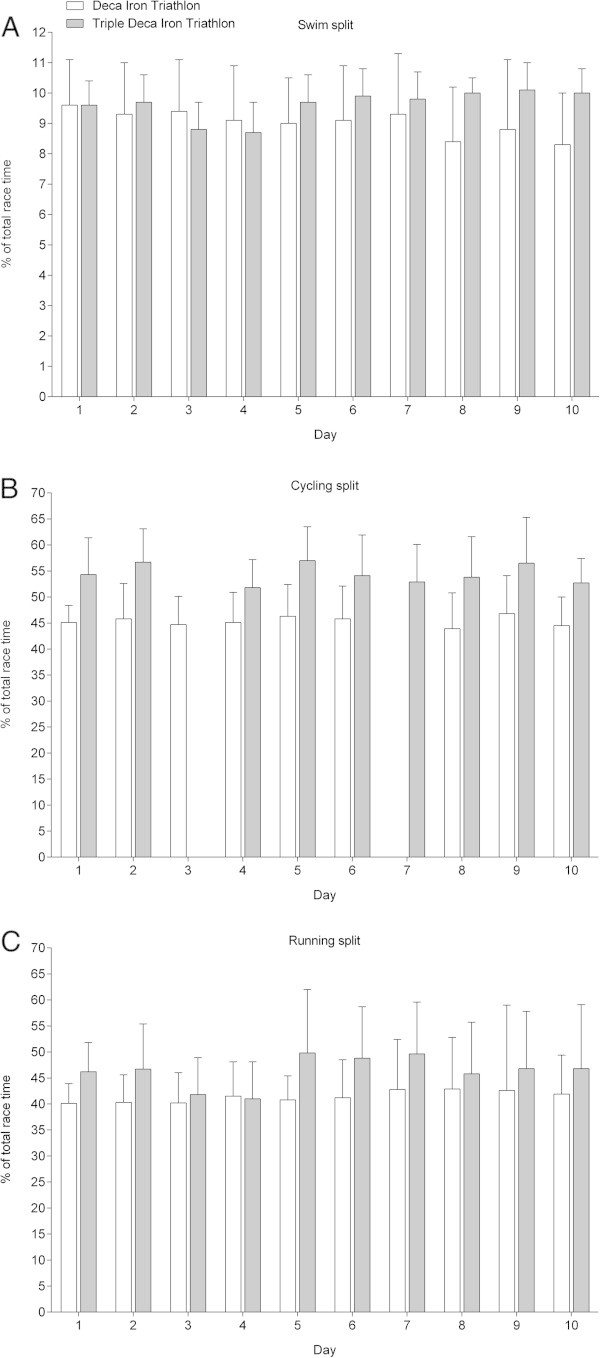
**Comparison of split and overall race times for Deca Iron ultra-triathletes (** ***i.e.*** **all ten race days) and Triple Deca Iron ultra-triathletes (** ***i.e.*** **Day 1-Day 10) expressed in percent of overall race time. (Panel A)** swimming, **(Panel B)** cycling, **(Panel C)** running. Values are means ± SD.

**Figure 7:**
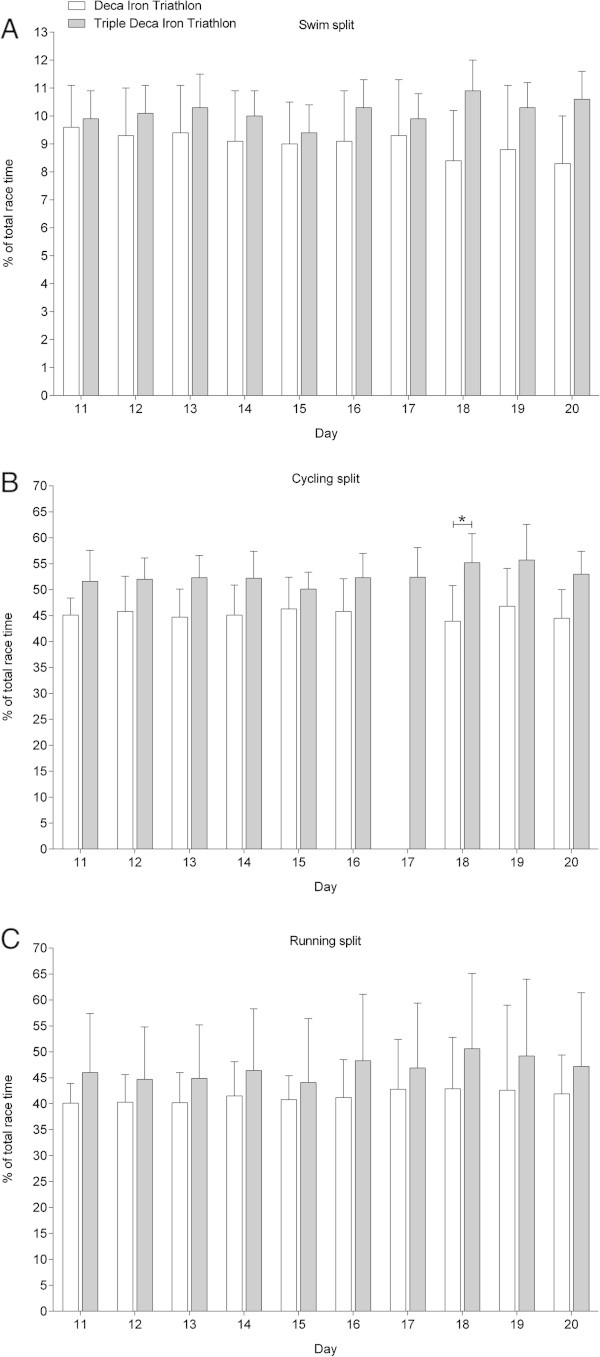
**Comparison of split and overall race times for Deca Iron ultra-triathletes (** ***i.e.*** **all ten race days) and Triple Deca Iron ultra-triathletes (** ***i.e.*** **Day 11-Day 20) expressed in percent of overall race time. (Panel A)** swimming, **(Panel B)** cycling, **(Panel C)** running. Values are means ± SD.

**Figure 8:**
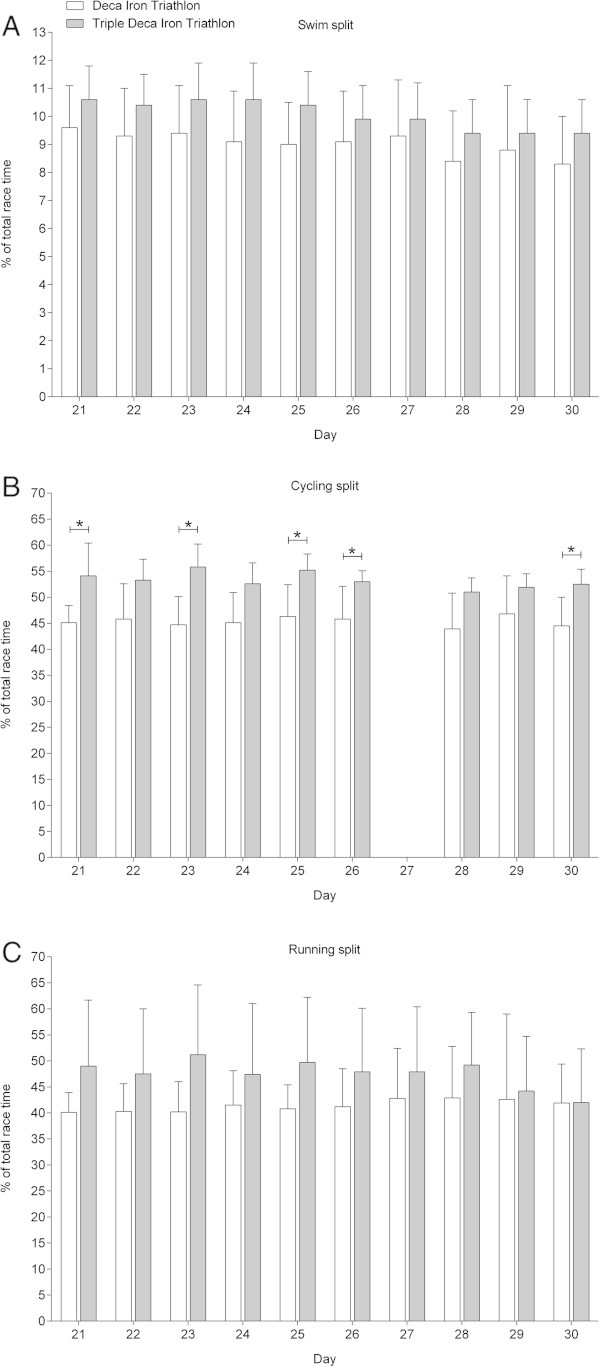
**Comparison of split and overall race times for Deca Iron ultra-triathletes (** ***i.e.*** **all ten race days) and Triple Deca Iron ultra-triathletes (** ***i.e.*** **Day 21-Day 30) expressed in percent of overall race time. (Panel A)** swimming, **(Panel B)** cycling, **(Panel C)** running. Values are means ± SD.

Figures 6, 7 and 8 show the comparisons in swimming, cycling and running expressed in percent of overall race time for Day 1–10 in the Deca Iron ultra-triathlon with Day 1–10 (Figure 6), Day 11–20 (Figure 7) and Day 21–30 (Figure 8) in the Triple Deca Iron ultra-triathlon. In the first ten days in the Triple Deca Iron ultra-triathlon, no differences were found compared to the ten days in the Deca Iron ultra-triathlon (Figure 6). When the second ten days in the Triple Deca Iron ultra-triathlon were compared to the ten days in the Deca Iron ultra-triathlon, the Deca Iron ultra-triathletes were relatively faster on Day 18 in cycling (Figure 7). On Day 21–30, the athletes in the Deca Iron ultra-triathlon were relatively faster in cycling on Day 1, Day 3, Day 5, Day 6 and Day 10 compared to the Triple Deca Iron ultra-triathletes (Figure 8).

**Figure 9:**
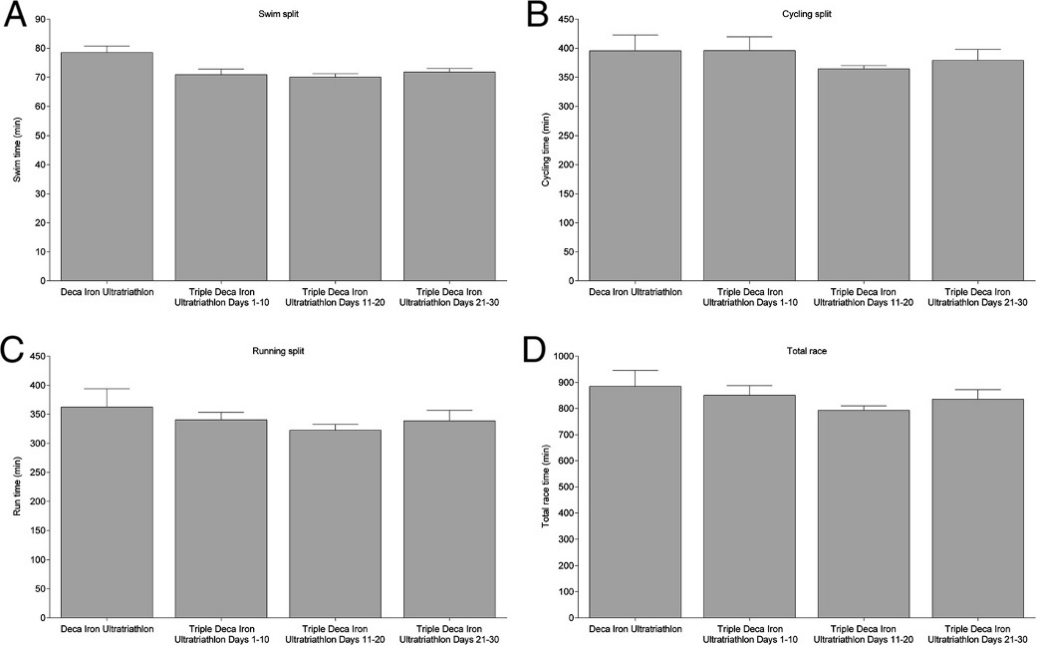
**Comparison of split and overall race times for Deca Iron ultra-triathletes with Day 1–10, Day 11–20 and Day 21–30 in Triple Deca Iron ultra-triathletes in absolute race times. (Panel A)** swimming, **(Panel B)** cycling, **(Panel C)** running, **(Panel D)** overall race times. Values are means ± SD.

Figure 9 presents the comparison in absolute times for swimming, cycling, running and overall race times of the ten days in the Deca Iron ultra-triathlon with Day 1–10, Day 11–20 and Day 21–30 in the Triple Deca Iron ultra-triathlon. The athletes in the Deca Iron ultra-triathlon were relatively slower in swimming compared to Day 1–10 (*p* < 0.001), Day 11–20 (*p* < 0.001) and Day 21–30 (*p* < 0.001) in the Triple Deca Iron ultra-triathlon. In cycling, Triple Deca Iron ultra-triathletes were relatively faster than Deca Iron ultra-triathletes considering Day 11–20 (*p* < 0.05) and Day 21–30 (*p* < 0.05). The Triple Deca Iron ultra-triathletes were relatively faster on Day 11–20 compared to Day 1–10 (*p* < 0.05). Considering running, Triple Deca Iron ultra-triathletes were relatively faster compared to Deca Iron ultra-triathletes on Day 11–20 (*p* < 0.05). And Triple Deca Iron ultra-triathletes were relatively faster on Day 11–20 compared to Day 1–10 (*p* < 0.05). For overall race time, Triple Deca Iron ultra-triathletes were relatively faster on Day 1–10 (*p* < 0.01) and Day 11–20 (*p* < 0.05) compared to Deca Iron ultra-triathletes. Again, Triple Deca Iron ultra-triathletes were relatively faster on Day 11–20 compared to Day 1–10 (*p* < 0.01).

**Figure 10:**
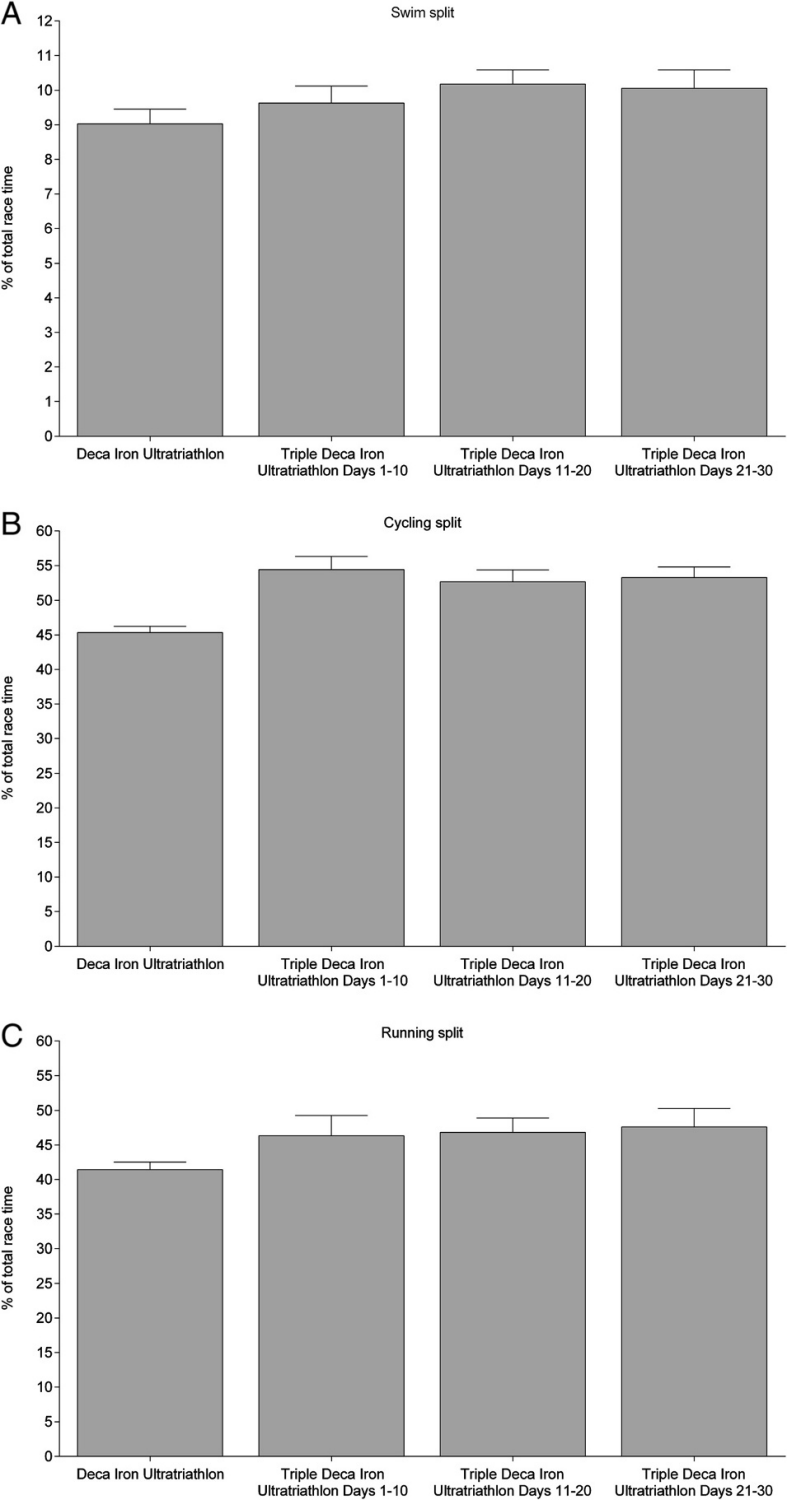
**Comparison of split and overall race times for Deca Iron ultra-triathletes with Day 1–10, Day 11–20 and Day 21–30 in Triple Deca Iron ultra-triathletes in percent of overall race time. (Panel A)** swimming, **(Panel B)** cycling, **(Panel C)** running. Values are means ± SD.

Figure 10 presents the comparison for swimming, cycling, and running in percent of overall race time of the ten days in the Deca Iron ultra-triathlon with Day 1–10, Day 11–20, and Day 21–30 in the Triple Deca Iron ultra-triathlon. Deca Iron ultra-triathletes were relatively faster in swimming compared to Triple Deca Iron ultra-triathletes on Day 11–20 (*p* < 0.01) and Day 21–30 (*p* < 0.001). In cycling, the Deca Iron ultra-triathletes were relatively faster compared to the Triple Deca Iron ultra-triathletes on Day 1–10 (*p* < 0.001), Day 11–20 (*p* < 0.001) and Day 21–30 (*p* < 0.001). Considering running, the Deca Iron ultra-triathletes were relatively faster on Day 1–10 (*p* < 0.01), Day 11–20 (*p* < 0.001) and Day 21–30 (*p* < 0.01) compared to the Triple Deca Iron ultra-triathletes.

## Discussion

This study intended to analyse the changes in performance over time for Triple Deca Iron ultra-triathletes and Deca Iron ultra-triathletes and it was hypothesized that performance would decrease in Deca Iron ultra-triathletes, but not in Triple Deca Iron ultra-triathletes. The most important finding was that the hypothesis was confirmed since performance decreased in Deca Iron ultra-triathletes but remained unchanged across time in Triple Deca Iron ultra-triathletes. The continuous decrease in performance in the Deca Iron ultra-triathletes confirms previous findings observed for Deca Iron ultra-triathletes competing in the 2006, 2007 and 2009 ‘World Challenge Deca Iron Triathlon’ held in Monterrey, Mexico (Herbst et al. 2011). The unchanged performance in swimming, cycling, running and overall race time in the Triple Deca Iron ultra-triathletes confirms very recent findings in an athlete completing 33 Ironman triathlons within 33 days where the athlete was able to maintain his performance in cycling, running and overall race time during the 33 days (Knechtle et al. 2014).

### The aspect of pacing strategy

An interesting observation was the difference in the comparison between absolute and relative performance. When the absolute daily performances of the Deca Iron ultra-triathletes were compared with the performances on Day 1–10, Day 11–20 and Day 21–30 of the Triple Iron ultra-triathletes, only minor differences were found. However, when the split performances were expressed relatively in percent of overall race times, Deca Iron ultra-triathletes were relatively faster in cycling in five of the ten days compared to the Triple Deca Iron ultra-triathletes in their last ten days. When the mean absolute performances of the ten days in the Deca Iron ultra-triathletes were compared to the performances on Day 1–10, Day 11–20 and Day 21–30 of the Triple Iron ultra-triathletes, the Deca Iron ultra-triathletes were slower in swimming, cycling and running compared to the specific segments of the Triple Deca Iron ultra-triathlon race. However, when the performances in the split disciplines was expressed in percent of overall race time, the Deca Iron ultra-triathletes were relatively faster in swimming, cycling and running compared to the three segments of the Triple Deca Iron ultra-triathlon race. A further interesting finding regarding absolute performances was that the Triple Iron ultra-triathletes were faster in the second segment of their race (*i.e.* Day 11–20) compared to their first segment (*i.e.* Day 1–10) in cycling, running, and overall performance. Generally, during ultra-endurance events (*i.e.* endurance performances lasting for longer than six hours), athletes tend to adopt a positive pacing strategy (*i.e.* the athlete’s speed gradually declines throughout the duration of the event) (Abbiss and Laursen 2008). The Triple Deca Iron ultra-triathletes, however, were able for a negative pacing in the first 20 days of the race. A potential explanation for the negative pacing (*i.e.* better performance in cycling, running and overall race times) could be the environmental conditions (*i.e.* unchanged course and habituation to the course), the habituation to the daily task for both athletes and support crews, and the stable weather conditions (*i.e.* no rain, constant air temperature of ~25–30°C and constant water temperature of 2 ~ 5°C in the first 21 days). Mean absolute performances in the second (*i.e.* Day 11–20) and third segment (*i.e.* Day 21–30) were not different. Most probably the athletes were then very experienced and negated the deterioration of the environmental conditions (*i.e.* decrease in air and water temperature).

Overall, the Triple Deca Iron ultra-triathletes showed an ‘even pacing’ during the 30 days in contrast to the Deca Iron ultra-triathletes with a ‘positive pacing’ (Abbiss and Laursen 2008) during their ten days. Most probably the successful Triple Deca Iron ultra-triathletes choose the right constant pace in cycling in the first days to be able to successfully finish the whole race. And they were even able to improve cycling and running speed in the second segment of the race. The athletes in the Triple Deca Iron ultra-triathlon might have become accustomed during the first days to the burden of a daily Ironman. They may have learned to adopt a pacing strategy to finish the race successfully. A potential explanation could be that the successful finishers in the Triple Deca Iron ultra-triathlon went slower in the cycling part in order to save energy for the running split which could be due to higher pre-race experience in Triple Deca Iron ultra-triathletes compared to Deca Iron ultra-triathletes. Alternatively, the Deca Iron ultra-triathletes went too fast in the first days in cycling leading to different problems such as muscular problems, loss in stored energy in the muscles, muscle soreness forcing them to go slower in the following days.

### The aspect of previous experience

An explanation for the differences in pacing strategy between the Deca Iron and the Triple Deca Iron ultra-triathletes could be the previous experience of the successful finishers. Previous experience is an important predictor variable in an ultra-triathlon (Herbst et al. 2011; Lepers et al. 2011). It has been shown that race time in a Triple Iron ultra-triathlon was highly predictive for race time in a Deca Iron ultra-triathlon (Herbst et al. 2011; Lepers et al. 2011). Overall race time in a Deca Iron ultra-triathlon might be predicted by the equation Deca Iron ultra-triathlon race time (min) = 5885 + 3.69 × race time in Triple Iron ultra-triathlon (minutes) (Lepers et al. 2011).

Since both the number and the personal best time in a Triple Iron ultra-triathlon were highly predictive for the performance in a Deca Iron ultra-triathlon (Herbst et al. 2011), we summarized for each finisher the number of completed Triple Iron ultra-triathlons with the personal best time with data available from the website of the International Ultra-Triathlon Association ( IUTA). In addition to the data of Triple Iron ultra-triathlon, we inserted also the data from Double Iron ultra-triathlon and longer ultra-triathlons for both finishers in the Deca Iron ultra-triathlon (Table 1) and in the Triple Deca Iron ultra-triathlon (Table 2). In accordance with previous reports (Herbst et al. 2011; Knechtle et al. 2011b; Lepers et al. 2011) the winner in the Deca Iron ultra-triathlon had the highest number of finishes in both Triple and Double Iron ultra-triathlon and the fastest personal best times in both Triple and Double Iron ultra-triathlon (Table 1) compared to the other finishers. For the fastest finishers in the Triple Deca Iron ultra-triathlon, however, both the numbers and the personal best times in both Triple and Double Iron ultra-triathlon seemed not of relevance (Table 2). The fastest three finishers had focussed more in their previous races on longer ultra-triathlon distances such as Deca Iron and Double Deca Iron ultra-triathlon. Similarly to the athletes in the Triple Deca Iron ultra-triathlon, the athlete completing 33 Ironman triathlons in 33 days in his self-paced race showed a broad experience in ultra-triathlon (Knechtle et al. 2014). He had finished 11 Double Iron ultra-triathlons with a personal best time of 21:48 h:min, six Triple Iron ultra-triathlons with a personal best time of 36:29 h:min and one Deca Iron ultra-triathlon within 297:42 h:min (Knechtle et al. 2014).

**Table 1:** **Number of finished ultra-triathlons with personal best time for Double Iron, Triple Iron and longer distances for finishers in the Deca Iron ultra-triathlon**

Rank	Number of finished Triple Iron ultra-triathlons	Personal best time in Triple Iron ultra-triathlon	Number of finished Double Iron ultra-triathlons	Personal best time in Double Iron ultra-triathlon	Other completed ultra-triathlons
**1**	24	37:18 h:min	22	22:57 h:min	3 Deca Iron with best time 240:55 h:min
**2**	-	-	2	25:10 h:min	-
**3**	1	47:19 h:min	4	26:34 h:min	1 Deca Iron in 285:40 h:min
**4**	1	55:29 h:min	-	-	-
**5**	-	-	-	-	-
**6**	1	46:45 h:min	-	-	-

**Table 2:** **Number of finished ultra-triathlons with personal best time for Double Iron, Triple Iron and longer distances for finishers in the Triple Deca Iron ultra-triathlon**

Rank	Number of finished Triple Iron ultra-triathlons	Personal best time Triple Iron ultra-triathlon	Number of finished Double Iron ultra-triathlons	Personal best time Double Iron ultra-triathlon	Other completed ultra-triathlons
**1**	-	-	4	25:47 h:min	1 Deca Iron in 268:12 h:min
**2**	1	40:14 h:min	4	23:55 h:min	1 Deca Iron in 222:17 h:min and 1 Double Deca Iron in 481:54 h:min
**3**	-	-	-	-	1 Double Deca Iron in 497:56 h:min
**4**	1	36:27 h:min	3	23:45 h:min	-
**5**	-	-	-	-	-
**6**	-	-	8	28:32 h:min	1 Deca Iron in 267:05 h:min
**7**	1	47:03 h:min	1	29:05 h:min	-
**8**	2	47:58 h:min	2	28:56 h:min	-

### Limitations

This study analysed the changes in performance in split and overall race times in Deca Iron and Triple Deca Iron ultra-triathletes. Unfortunately, aspects such as nutrition (Dempster et al. 2013; Knechtle et al. 2008b), fluid metabolism (Knechtle et al. 2008b), sleep and sleep deprivation (Knechtle et al. 2012b; Lahart et al. 2013), recovery (Neubauer et al. 2008), pain tolerance (Freund et al. 2013), association between anthropometry and performance (Knechtle et al. 2010), changes in body composition (Herbst et al. 2011; Knechtle et al. 2008a, [b]; Mueller et al. 2013; Schütz et al. 2013), and overuse injuries of the lower limbs (Freund et al. 2012) were not included.

## Conclusions

This study showed that performance decreased linearly across days for Deca Iron ultra-triathletes (*i.e.* positive pacing) while performance remained unchanged across days for Triple Deca Iron ultra-triathletes (*i.e.* even pacing). To be successful in a Triple Deca Iron ultra-triathlon, a high number and a fast personal best time in ultra-triathlons shorter than the Triple Deca Iron ultra-triathlon seem mandatory. We assume that ultra-triathletes successfully competing in longer races than a Deca Iron ultra-triathlon such as a Triple Deca Iron ultra-triathlon need to gain experience in the long ultra-triathlon distances such as a Deca Iron ultra-triathlon.
